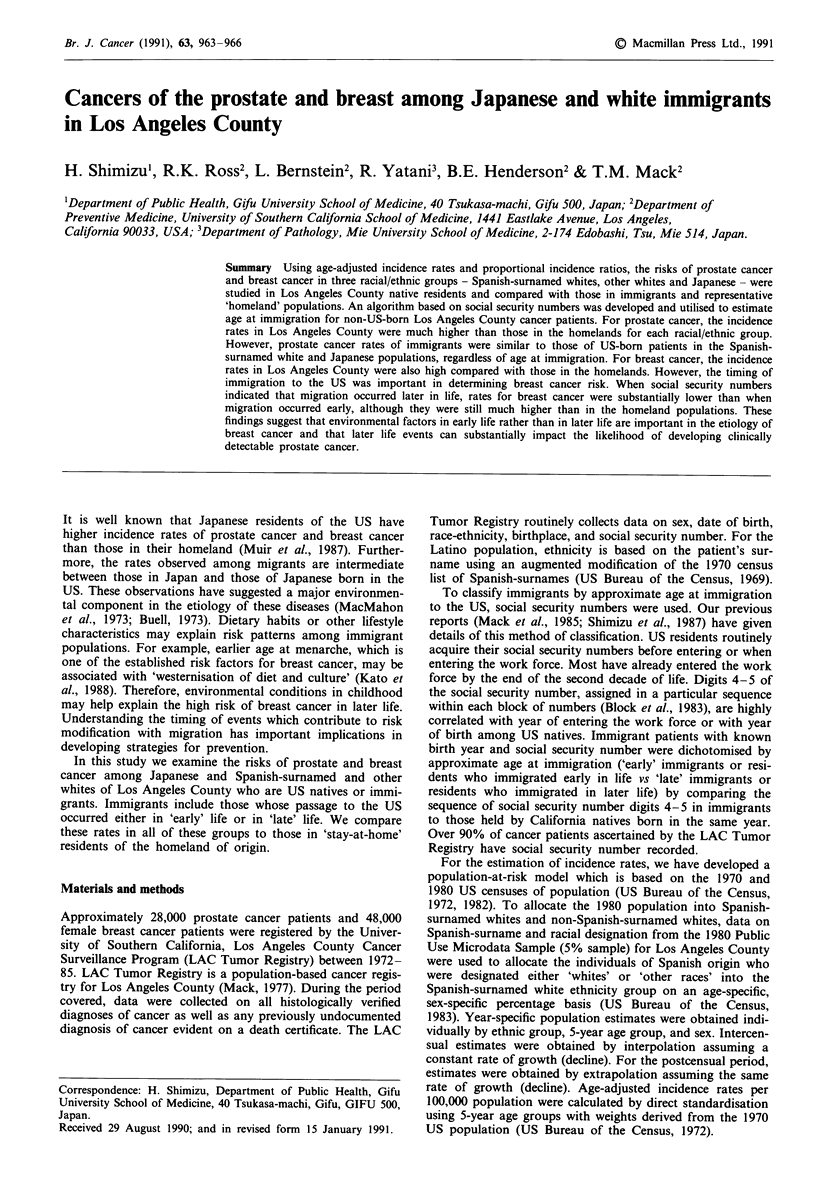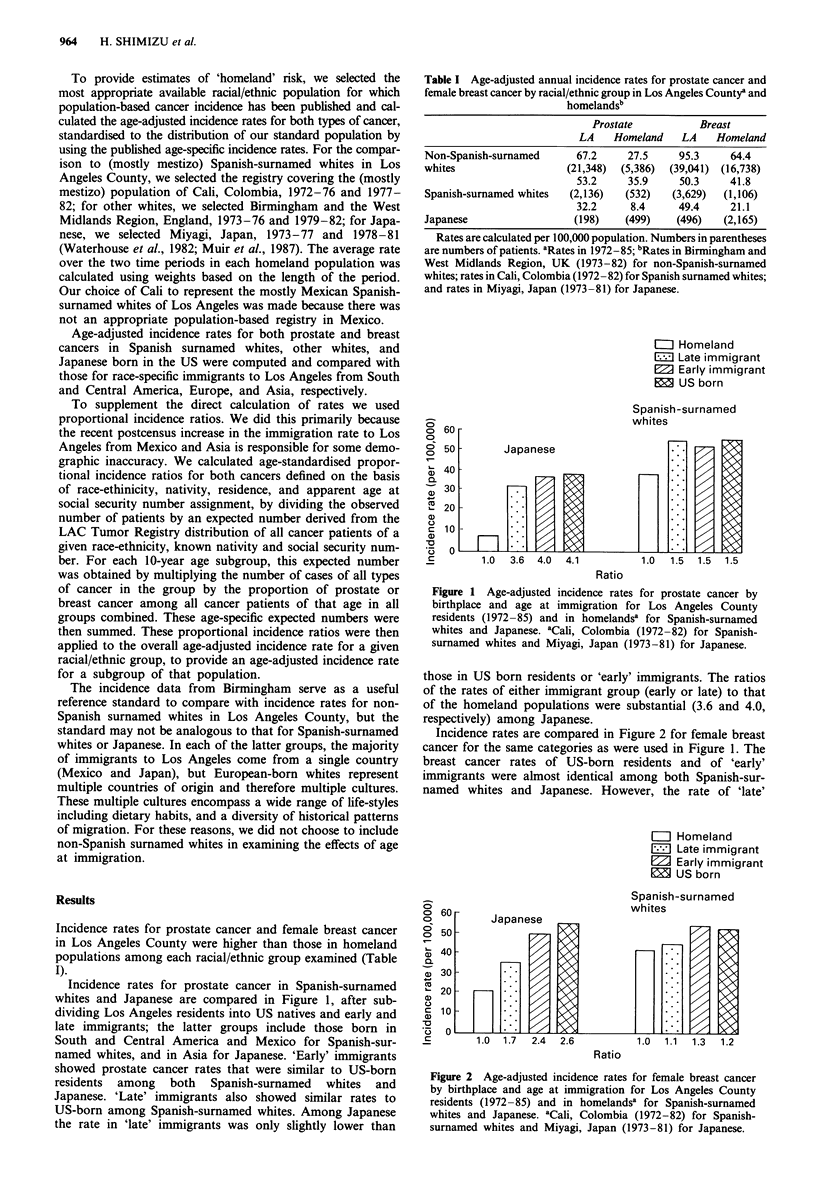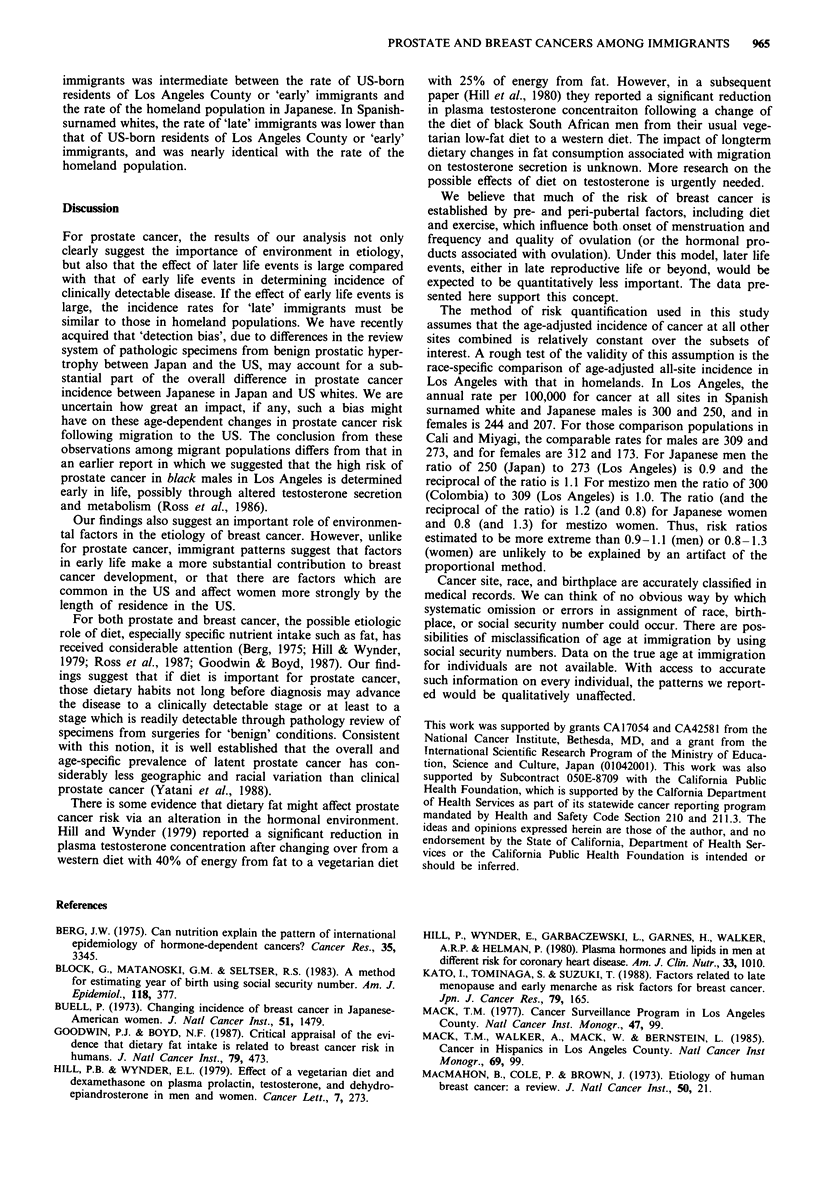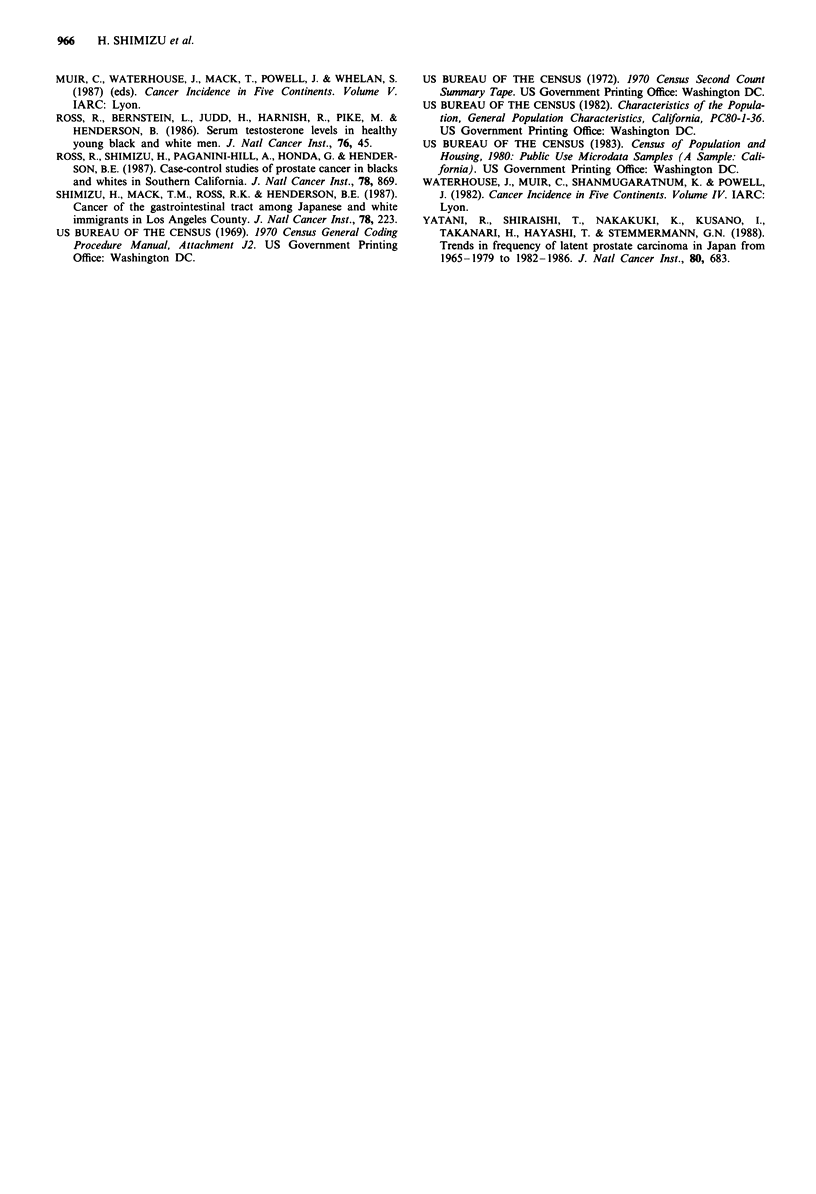# Cancers of the prostate and breast among Japanese and white immigrants in Los Angeles County.

**DOI:** 10.1038/bjc.1991.210

**Published:** 1991-06

**Authors:** H. Shimizu, R. K. Ross, L. Bernstein, R. Yatani, B. E. Henderson, T. M. Mack

**Affiliations:** Department of Public Health, Gifu University School of Medicine, Japan.

## Abstract

Using age-adjusted incidence rates and proportional incidence ratios, the risks of prostate cancer and breast cancer in three racial/ethnic groups - Spanish-surnamed whites, other whites and Japanese - were studied in Los Angeles County native residents and compared with those in immigrants and representative 'homeland' populations. An algorithm based on social security numbers was developed and utilised to estimate age at immigration for non-US-born Los Angeles County cancer patients. For prostate cancer, the incidence rates in Los Angeles County were much higher than those in the homelands for each racial/ethnic group. However, prostate cancer rates of immigrants were similar to those of US-born patients in the Spanish-surnamed white and Japanese populations, regardless of age at immigration. For breast cancer, the incidence rates in Los Angeles County were also high compared with those in the homelands. However, the timing of immigration to the US was important in determining breast cancer risk. When social security numbers indicated that migration occurred later in life, rates for breast cancer were substantially lower than when migration occurred early, although they were still much higher than in the homeland populations. These findings suggest that environmental factors in early life rather than in later life are important in the etiology of breast cancer and that later life events can substantially impact the likelihood of developing clinically detectable prostate cancer.


Br. J. Cancer (1991), 63, 963-966 © Macmillan Press Ltd., 1991

### Cancers of the prostate and breast among Japanese and white immigrants in Los Angeles County

H. Shimizu[1], R.K. Ross[2], L. Bernstein[2], R. Yatani[3], B.E. Henderson[2] & T.M. Mack[2]

[1]*Department of Public Health, Gifu University School of Medicine, 40 Tsukasa-machi, Gifu 500, Japan;* [2]*Department of Preventive Medicine, University of Southern California School of Medicine, 1441 Eastlake Avenue, Los Angeles, California 90033, USA;* [3]*Department of Pathology, Mie University School of Medicine, 2-174 Edobashi, Tsu, Mie 514, Japan.*

**Summary** Using age-adjusted incidence rates and proportional incidence ratios, the risks of prostate cancer and breast cancer in three racial/ethnic groups – Spanish-surnamed whites, other whites and Japanese – were studied in Los Angeles County native residents and compared with those in immigrants and representative 'homeland' populations. An algorithm based on social security numbers was developed and utilised to estimate age at immigration for non-US-born Los Angeles County cancer patients. For prostate cancer, the incidence rates in Los Angeles County were much higher than those in the homelands for each racial/ethnic group. However, prostate cancer rates of immigrants were similar to those of US-born patients in the Spanish-surnamed white and Japanese populations, regardless of age at immigration. For breast cancer, the incidence rates in Los Angeles County were also high compared with those in the homelands. However, the timing of immigration to the US was important in determining breast cancer risk. When social security numbers indicated that migration occurred later in life, rates for breast cancer were substantially lower than when migration occurred early, although they were still much higher than in the homeland populations. These findings suggest that environmental factors in early life rather than in later life are important in the etiology of breast cancer and that later life events can substantially impact the likelihood of developing clinically detectable prostate cancer.

It is well known that Japanese residents of the US have higher incidence rates of prostate cancer and breast cancer than those in their homeland (Muir *et al.*, 1987). Furthermore, the rates observed among migrants are intermediate between those in Japan and those of Japanese born in the US. These observations have suggested a major environmental component in the etiology of these diseases (MacMahon *et al.*, 1973; Buell, 1973). Dietary habits or other lifestyle characteristics may explain risk patterns among immigrant populations. For example, earlier age at menarche, which is one of the established risk factors for breast cancer, may be associated with 'westernisation of diet and culture' (Kato *et al.*, 1988). Therefore, environmental conditions in childhood may help explain the high risk of breast cancer in later life. Understanding the timing of events which contribute to risk modification with migration has important implications in developing strategies for prevention.

In this study we examine the risks of prostate and breast cancer among Japanese and Spanish-surnamed and other whites of Los Angeles County who are US natives or immigrants. Immigrants include those whose passage to the US occurred either in 'early' life or in 'late' life. We compare these rates in all of these groups to those in 'stay-at-home' residents of the homeland of origin.

### Materials and methods

Approximately 28,000 prostate cancer patients and 48,000 female breast cancer patients were registered by the University of Southern California, Los Angeles County Cancer Surveillance Program (LAC Tumor Registry) between 1972–85. LAC Tumor Registry is a population-based cancer registry for Los Angeles County (Mack, 1977). During the period covered, data were collected on all histologically verified diagnoses of cancer as well as any previously undocumented diagnosis of cancer evident on a death certificate. The LAC Tumor Registry routinely collects data on sex, date of birth, race-ethnicity, birthplace, and social security number. For the Latino population, ethnicity is based on the patient's surname using an augmented modification of the 1970 census list of Spanish-surnames (US Bureau of the Census, 1969).

To classify immigrants by approximate age at immigration to the US, social security numbers were used. Our previous reports (Mack *et al.*, 1985; Shimizu *et al.*, 1987) have given details of this method of classification. US residents routinely acquire their social security numbers before entering or when entering the work force. Most have already entered the work force by the end of the second decade of life. Digits 4–5 of the social security number, assigned in a particular sequence within each block of numbers (Block *et al.*, 1983), are highly correlated with year of entering the work force or with year of birth among US natives. Immigrant patients with known birth year and social security number were dichotomised by approximate age at immigration ('early' immigrants or residents who immigrated early in life *vs* 'late' immigrants or residents who immigrated in later life) by comparing the sequence of social security number digits 4–5 in immigrants to those held by California natives born in the same year. Over 90% of cancer patients ascertained by the LAC Tumor Registry have social security number recorded.

For the estimation of incidence rates, we have developed a population-at-risk model which is based on the 1970 and 1980 US censuses of population (US Bureau of the Census, 1972, 1982). To allocate the 1980 population into Spanish-surnamed whites and non-Spanish-surnamed whites, data on Spanish-surname and racial designation from the 1980 Public Use Microdata Sample (5% sample) for Los Angeles County were used to allocate the individuals of Spanish origin who were designated either 'whites' or 'other races' into the Spanish-surnamed white ethnicity group on an age-specific, sex-specific percentage basis (US Bureau of the Census, 1983). Year-specific population estimates were obtained individually by ethnic group, 5-year age group, and sex. Intercensual estimates were obtained by interpolation assuming a constant rate of growth (decline). For the postcensual period, estimates were obtained by extrapolation assuming the same rate of growth (decline). Age-adjusted incidence rates per 100,000 population were calculated by direct standardisation using 5-year age groups with weights derived from the 1970 US population (US Bureau of the Census, 1972).

Correspondence: H. Shimizu, Department of Public Health, Gifu University School of Medicine, 40 Tsukasa-machi, Gifu, GIFU 500, Japan.
Received 29 August 1990; and in revised form 15 January 1991.

To provide estimates of 'homeland' risk, we selected the most appropriate available racial/ethnic population for which population-based cancer incidence has been published and calculated the age-adjusted incidence rates for both types of cancer, standardised to the distribution of our standard population by using the published age-specific incidence rates. For the comparison to (mostly mestizo) Spanish-surnamed whites in Los Angeles County, we selected the registry covering the (mostly mestizo) population of Cali, Colombia, 1972–76 and 1977–82; for other whites, we selected Birmingham and the West Midlands Region, England, 1973–76 and 1979–82; for Japanese, we selected Miyagi, Japan, 1973–77 and 1978–81 (Waterhouse *et al.*, 1982; Muir *et al.*, 1987). The average rate over the two time periods in each homeland population was calculated using weights based on the length of the period. Our choice of Cali to represent the mostly Mexican Spanish-surnamed whites of Los Angeles was made because there was not an appropriate population-based registry in Mexico.

Age-adjusted incidence rates for both prostate and breast cancers in Spanish surnamed whites, other whites, and Japanese born in the US were computed and compared with those for race-specific immigrants to Los Angeles from South and Central America, Europe, and Asia, respectively.

To supplement the direct calculation of rates we used proportional incidence ratios. We did this primarily because the recent postcensus increase in the immigration rate to Los Angeles from Mexico and Asia is responsible for some demographic inaccuracy. We calculated age-standardised proportional incidence ratios for both cancers defined on the basis of race-ethinicity, nativity, residence, and apparent age at social security number assignment, by dividing the observed number of patients by an expected number derived from the LAC Tumor Registry distribution of all cancer patients of a given race-ethnicity, known nativity and social security number. For each 10-year age subgroup, this expected number was obtained by multiplying the number of cases of all types of cancer in the group by the proportion of prostate or breast cancer among all cancer patients of that age in all groups combined. These age-specific expected numbers were then summed. These proportional incidence ratios were then applied to the overall age-adjusted incidence rate for a given racial/ethnic group, to provide an age-adjusted incidence rate for a subgroup of that population.

The incidence data from Birmingham serve as a useful reference standard to compare with incidence rates for non-Spanish surnamed whites in Los Angeles County, but the standard may not be analogous to that for Spanish-surnamed whites or Japanese. In each of the latter groups, the majority of immigrants to Los Angeles come from a single country (Mexico and Japan), but European-born whites represent multiple countries of origin and therefore multiple cultures. These multiple cultures encompass a wide range of life-styles including dietary habits, and a diversity of historical patterns of migration. For these reasons, we did not choose to include non-Spanish surnamed whites in examining the effects of age at immigration.

### Results

Incidence rates for prostate cancer and female breast cancer in Los Angeles County were higher than those in homeland populations among each racial/ethnic group examined (Table I).

Incidence rates for prostate cancer in Spanish-surnamed whites and Japanese are compared in Figure 1, after subdividing Los Angeles residents into US natives and early and late immigrants; the latter groups include those born in South and Central America and Mexico for Spanish-surnamed whites, and in Asia for Japanese. 'Early' immigrants showed prostate cancer rates that were similar to US-born residents among both Spanish-surnamed whites and Japanese. 'Late' immigrants also showed similar rates to US-born among Spanish-surnamed whites. Among Japanese the rate in 'late' immigrants was only slightly lower than those in US born residents or 'early' immigrants. The ratios of the rates of either immigrant group (early or late) to that of the homeland populations were substantial (3.6 and 4.0, respectively) among Japanese.

Incidence rates are compared in Figure 2 for female breast cancer for the same categories as were used in Figure 1. The breast cancer rates of US-born residents and of 'early' immigrants were almost identical among both Spanish-surnamed whites and Japanese. However, the rate of 'late'

**Table I** Age-adjusted annual incidence rates for prostate cancer and female breast cancer by racial/ethnic group in Los Angeles County[a] and homelands[b]

| | *Prostate* | | *Breast* | |
|---|---|---|---|---|
| | *LA* | *Homeland* | *LA* | *Homeland* |
| Non-Spanish-surnamed whites | 67.2 (21,348) | 27.5 (5,386) | 95.3 (39,041) | 64.4 (16,738) |
| Spanish-surnamed whites | 53.2 (2,136) | 35.9 (532) | 50.3 (3,629) | 41.8 (1,106) |
| Japanese | 32.2 (198) | 8.4 (499) | 49.4 (496) | 21.1 (2,165) |

Rates are calculated per 100,000 population. Numbers in parentheses are numbers of patients. [a]Rates in 1972–85; [b]Rates in Birmingham and West Midlands Region, UK (1973–82) for non-Spanish-surnamed whites; rates in Cali, Colombia (1972–82) for Spanish surnamed whites; and rates in Miyagi, Japan (1973–81) for Japanese.

**Figure 1** Age-adjusted incidence rates for prostate cancer by birthplace and age at immigration for Los Angeles County residents (1972–85) and in homelands[a] for Spanish-surnamed whites and Japanese. [a]Cali, Colombia (1972–82) for Spanish-surnamed whites and Miyagi, Japan (1973–81) for Japanese.

**Figure 2** Age-adjusted incidence rates for female breast cancer by birthplace and age at immigration for Los Angeles County residents (1972–85) and in homelands[a] for Spanish-surnamed whites and Japanese. [a]Cali, Colombia (1972–82) for Spanish-surnamed whites and Miyagi, Japan (1973–81) for Japanese.

immigrants was intermediate between the rate of US-born residents of Los Angeles County or 'early' immigrants and the rate of the homeland population in Japanese. In Spanish-surnamed whites, the rate of 'late' immigrants was lower than that of US-born residents of Los Angeles County or 'early' immigrants, and was nearly identical with the rate of the homeland population.

### Discussion

For prostate cancer, the results of our analysis not only clearly suggest the importance of environment in etiology, but also that the effect of later life events is large compared with that of early life events in determining incidence of clinically detectable disease. If the effect of early life events is large, the incidence rates for 'late' immigrants must be similar to those in homeland populations. We have recently acquired that 'detection bias', due to differences in the review system of pathologic specimens from benign prostatic hypertrophy between Japan and the US, may account for a substantial part of the overall difference in prostate cancer incidence between Japanese in Japan and US whites. We are uncertain how great an impact, if any, such a bias might have on these age-dependent changes in prostate cancer risk following migration to the US. The conclusion from these observations among migrant populations differs from that in an earlier report in which we suggested that the high risk of prostate cancer in *black* males in Los Angeles is determined early in life, possibly through altered testosterone secretion and metabolism (Ross *et al.*, 1986).

Our findings also suggest an important role of environmental factors in the etiology of breast cancer. However, unlike for prostate cancer, immigrant patterns suggest that factors in early life make a more substantial contribution to breast cancer development, or that there are factors which are common in the US and affect women more strongly by the length of residence in the US.

For both prostate and breast cancer, the possible etiologic role of diet, especially specific nutrient intake such as fat, has received considerable attention (Berg, 1975; Hill & Wynder, 1979; Ross *et al.*, 1987; Goodwin & Boyd, 1987). Our findings suggest that if diet is important for prostate cancer, those dietary habits not long before diagnosis may advance the disease to a clinically detectable stage or at least to a stage which is readily detectable through pathology review of specimens from surgeries for 'benign' conditions. Consistent with this notion, it is well established that the overall and age-specific prevalence of latent prostate cancer has considerably less geographic and racial variation than clinical prostate cancer (Yatani *et al.*, 1988).

There is some evidence that dietary fat might affect prostate cancer risk via an alteration in the hormonal environment. Hill and Wynder (1979) reported a significant reduction in plasma testosterone concentration after changing over from a western diet with 40% of energy from fat to a vegetarian diet with 25% of energy from fat. However, in a subsequent paper (Hill *et al.*, 1980) they reported a significant reduction in plasma testosterone concentraiton following a change of the diet of black South African men from their usual vegetarian low-fat diet to a western diet. The impact of longterm dietary changes in fat consumption associated with migration on testosterone secretion is unknown. More research on the possible effects of diet on testosterone is urgently needed.

We believe that much of the risk of breast cancer is established by pre- and peri-pubertal factors, including diet and exercise, which influence both onset of menstruation and frequency and quality of ovulation (or the hormonal products associated with ovulation). Under this model, later life events, either in late reproductive life or beyond, would be expected to be quantitatively less important. The data presented here support this concept.

The method of risk quantification used in this study assumes that the age-adjusted incidence of cancer at all other sites combined is relatively constant over the subsets of interest. A rough test of the validity of this assumption is the race-specific comparison of age-adjusted all-site incidence in Los Angeles with that in homelands. In Los Angeles, the annual rate per 100,000 for cancer at all sites in Spanish surnamed white and Japanese males is 300 and 250, and in females is 244 and 207. For those comparison populations in Cali and Miyagi, the comparable rates for males are 309 and 273, and for females are 312 and 173. For Japanese men the ratio of 250 (Japan) to 273 (Los Angeles) is 0.9 and the reciprocal of the ratio is 1.1 For mestizo men the ratio of 300 (Colombia) to 309 (Los Angeles) is 1.0. The ratio (and the reciprocal of the ratio) is 1.2 (and 0.8) for Japanese women and 0.8 (and 1.3) for mestizo women. Thus, risk ratios estimated to be more extreme than 0.9–1.1 (men) or 0.8–1.3 (women) are unlikely to be explained by an artifact of the proportional method.

Cancer site, race, and birthplace are accurately classified in medical records. We can think of no obvious way by which systematic omission or errors in assignment of race, birthplace, or social security number could occur. There are possibilities of misclassification of age at immigration by using social security numbers. Data on the true age at immigration for individuals are not available. With access to accurate such information on every individual, the patterns we reported would be qualitatively unaffected.

This work was supported by grants CA17054 and CA42581 from the National Cancer Institute, Bethesda, MD, and a grant from the International Scientific Research Program of the Ministry of Education, Science and Culture, Japan (01042001). This work was also supported by Subcontract 050E-8709 with the California Public Health Foundation, which is supported by the Calfornia Department of Health Services as part of its statewide cancer reporting program mandated by Health and Safety Code Section 210 and 211.3. The ideas and opinions expressed herein are those of the author, and no endorsement by the State of California, Department of Health Services or the California Public Health Foundation is intended or should be inferred.

### References

BERG, J.W. (1975). Can nutrition explain the pattern of international epidemiology of hormone-dependent cancers? *Cancer Res.*, **35**, 3345.

BLOCK, G., MATANOSKI, G.M. & SELTSER, R.S. (1983). A method for estimating year of birth using social security number. *Am. J. Epidemiol.*, **118**, 377.

BUELL, P. (1973). Changing incidence of breast cancer in Japanese-American women. *J. Natl Cancer Inst.*, **51**, 1479.

GOODWIN, P.J. & BOYD, N.F. (1987). Critical appraisal of the evidence that dietary fat intake is related to breast cancer risk in humans. *J. Natl Cancer Inst.*, **79**, 473.

HILL, P.B. & WYNDER, E.L. (1979). Effect of a vegetarian diet and dexamethasone on plasma prolactin, testosterone, and dehydroepiandrosterone in men and women. *Cancer Lett.*, **7**, 273.

HILL, P., WYNDER, E., GARBACZEWSKI, L., GARNES, H., WALKER, A.R.P. & HELMAN, P. (1980). Plasma hormones and lipids in men at different risk for coronary heart disease. *Am. J. Clin. Nutr.*, **33**, 1010.

KATO, I., TOMINAGA, S. & SUZUKI, T. (1988). Factors related to late menopause and early menarche as risk factors for breast cancer. *Jpn. J. Cancer Res.*, **79**, 165.

MACK, T.M. (1977). Cancer Surveillance Program in Los Angeles County. *Natl Cancer Inst. Monogr.*, **47**, 99.

MACK, T.M., WALKER, A., MACK, W. & BERNSTEIN, L. (1985). Cancer in Hispanics in Los Angeles County. *Natl Cancer Inst Monogr.*, **69**, 99.

MACMAHON, B., COLE, P. & BROWN, J. (1973). Etiology of human breast cancer: a review. *J. Natl Cancer Inst.*, **50**, 21.

MUIR, C., WATERHOUSE, J., MACK, T., POWELL, J. & WHELAN, S. (1987) (eds). *Cancer Incidence in Five Continents. Volume V.* IARC: Lyon.

ROSS, R., BERNSTEIN, L., JUDD, H., HARNISH, R., PIKE, M. & HENDERSON, B. (1986). Serum testosterone levels in healthy young black and white men. *J. Natl Cancer Inst.*, **76**, 45.

ROSS, R., SHIMIZU, H., PAGANINI-HILL, A., HONDA, G. & HENDERSON, B.E. (1987). Case-control studies of prostate cancer in blacks and whites in Southern California. *J. Natl Cancer Inst.*, **78**, 869.

SHIMIZU, H., MACK, T.M., ROSS, R.K. & HENDERSON, B.E. (1987). Cancer of the gastrointestinal tract among Japanese and white immigrants in Los Angeles County. *J. Natl Cancer Inst.*, **78**, 223.

US BUREAU OF THE CENSUS (1969). *1970 Census General Coding Procedure Manual, Attachment J2.* US Government Printing Office: Washington DC.

US BUREAU OF THE CENSUS (1972). *1970 Census Second Count Summary Tape.* US Government Printing Office: Washington DC.

US BUREAU OF THE CENSUS (1982). *Characteristics of the Population, General Population Characteristics, California, PC80-1-36.* US Government Printing Office: Washington DC.

US BUREAU OF THE CENSUS (1983). *Census of Population and Housing, 1980: Public Use Microdata Samples (A Sample: California).* US Government Printing Office: Washington DC.

WATERHOUSE, J., MUIR, C., SHANMUGARATNUM, K. & POWELL, J. (1982). *Cancer Incidence in Five Continents. Volume IV.* IARC: Lyon.

YATANI, R., SHIRAISHI, T., NAKAKUKI, K., KUSANO, I., TAKANARI, H., HAYASHI, T. & STEMMERMANN, G.N. (1988). Trends in frequency of latent prostate carcinoma in Japan from 1965-1979 to 1982-1986. *J. Natl Cancer Inst.*, **80**, 683.